# Cosmic radiation exposure and persistent cognitive dysfunction

**DOI:** 10.1038/srep34774

**Published:** 2016-10-10

**Authors:** Vipan K. Parihar, Barrett D. Allen, Chongshan Caressi, Stephanie Kwok, Esther Chu, Katherine K. Tran, Nicole N. Chmielewski, Erich Giedzinski, Munjal M. Acharya, Richard A. Britten, Janet E. Baulch, Charles L. Limoli

**Affiliations:** 1 Department of Radiation Oncology, University of California, Irvine, CA 92697-2695, USA; 2 Department of Radiation Oncology, Eastern Virginia Medical School, Norfolk, VA 23507, USA.

## Abstract

The Mars mission will result in an inevitable exposure to cosmic radiation that has been shown to cause cognitive impairments in rodent models, and possibly in astronauts engaged in deep space travel. Of particular concern is the potential for cosmic radiation exposure to compromise critical decision making during normal operations or under emergency conditions in deep space. Rodents exposed to cosmic radiation exhibit persistent hippocampal and cortical based performance decrements using six independent behavioral tasks administered between separate cohorts 12 and 24 weeks after irradiation. Radiation-induced impairments in spatial, episodic and recognition memory were temporally coincident with deficits in executive function and reduced rates of fear extinction and elevated anxiety. Irradiation caused significant reductions in dendritic complexity, spine density and altered spine morphology along medial prefrontal cortical neurons known to mediate neurotransmission interrogated by our behavioral tasks. Cosmic radiation also disrupted synaptic integrity and increased neuroinflammation that persisted more than 6 months after exposure. Behavioral deficits for individual animals correlated significantly with reduced spine density and increased synaptic puncta, providing quantitative measures of risk for developing cognitive impairment. Our data provide additional evidence that deep space travel poses a real and unique threat to the integrity of neural circuits in the brain.

The exploration of space presents countless challenges to the ingenuity of humankind. Vast distances separate our planet from those within and beyond our solar system and necessitate further advances in engineering to minimize the deep space travel time and in biology to ameliorate as many of the adverse effects of prolonged space travel as possible. While many threats to the success of such extraterrestrial missions have been popularized in the media and entertainment industries, one area that has not received such attention are the human health risks associated with cosmic radiation exposure. As NASA plans a mission to Mars, astronauts will inevitably be exposed to low fluences of highly energetic and fully ionized nuclei that define the spectrum of galactic cosmic rays (GCR)^1^,^2^,^3^. Charged particles that represent the GCR are a component of cosmic radiation that is deflected from the surface of the Earth by its protective magnetosphere. Due to their high energy, multiple charged particle species can penetrate the hull of a spacecraft and tissues of the body depositing a trail of dense ionizations along particle trajectories^3^. In the body, the ionization events resulting from these interactions damage a variety of critical molecular targets, producing complex lesions that compromise cellular repair processes and protract the recovery of irradiated tissues. Recovery from cosmic radiation injury is further confounded by secondary ionizations caused by delta rays that emanate from primary particle tracks, considerably increasing the range and amount of cellular damage^4^,^5^.

NASA and international space agencies have recognized the potential health concerns associated with cosmic radiation exposure^6^, and based on recent evidence derived from rodent models have an increased awareness of the potential neurocognitive complications that could compromise mission critical activities or long term cognitive health^7^. Despite our long-standing knowledge that patients subjected to cranial radiotherapy for the control of brain malignancies develop severe and progressive cognitive deficits^8^,^9^, the total doses and radiation types used in the clinic differ significantly from those encountered in space. Compelling evidence has now demonstrated the adverse effects of space-relevant fluences of charged particles on cognition^7^,^10^,^11^,^12^,^13^,^14^,^15^, and our studies, have linked functional behavioral decrements to the erosion of neuronal structure and synaptic integrity in specific regions of the brain^7^,^16^. Importantly, these changes were found to persist 6 weeks following acute exposure of rodents to charged particles, and showed little or no signs of recovery, regeneration or repair^7^. Here, we extend our studies longer term and show convincingly that very low doses of charged particles can compromise cognitive performance at not only 12, but 24 weeks after acute exposure, effects that are associated with reductions in dendritic complexity, changes in synaptic protein levels and elevations in neuroinflammation.

## Results

### Cosmic radiation causes long-term cognitive dysfunction

**Figure 1:**
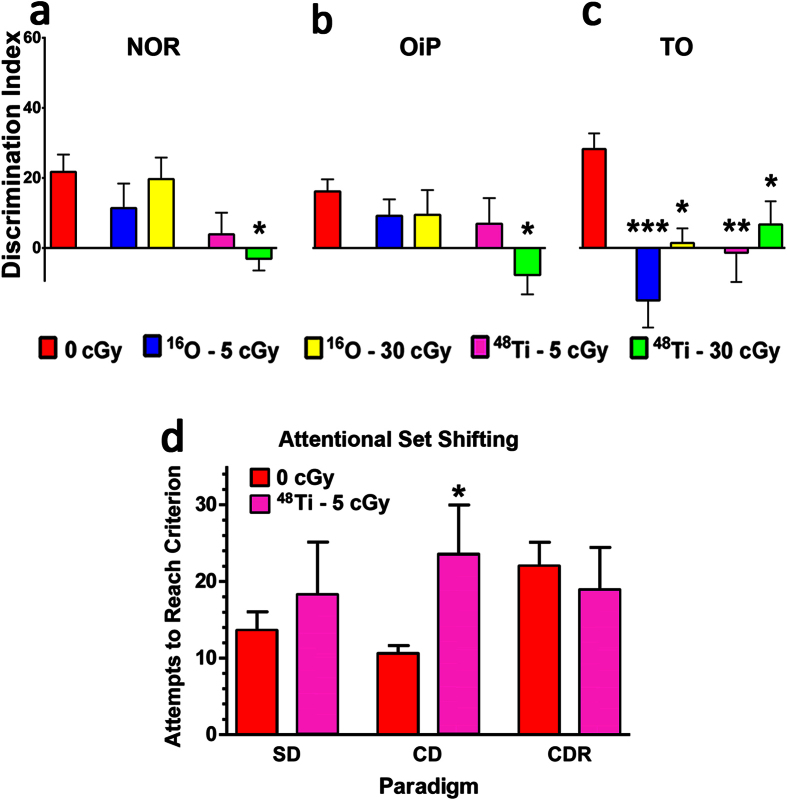
Cognitive deficits evaluated 12 weeks after cosmic radiation exposure. (**a**) Analysis of preference for novelty on a **N**ovel **O**bject **R**ecognition (NOR) task shows that 30 cGy ^48^Ti particle irradiation significantly reduced recognition memory. (**b**) Performance on an **O**bject **i**n **P**lace (OiP) task shows decrements in spatial memory retention for mice exposed to 30 cGy ^48^Ti particles as manifested in a reduced preference to explore an object found in a novel location. (**c**) All ^48^Ti and ^16^O irradiations significantly impaired recency memory as evident by a reduced preference for the less recently explored object in the **T**emporal **O**rder task (TO). *P < 0.05, **P < 0.01, ***P < 0.001; one-way ANOVA followed by Bonferroni’s multiple comparison post hoc analysis. (**d**) Attentional set shifting performance of adult male Wistar rats at 12 weeks post irradiation. Number of attempts required to reach criterion in the Simple Discrimination (SD); Compound Discrimination (CD) and Compound Discrimination Reversal (CDR) paradigms. Graphs show means ± SEM for control rats or rats exposed to 5 cGy 1 GeV/n ^48^Ti ions. *P = 0.048 (Mann-Whitney, compared to respective control values).

To assess the functional consequences of cosmic radiation exposure on the brain, we behaviorally tested mice 12 weeks after exposure to ^48^Ti or ^16^O (Fig. 1). While impairments on the novel object recognition (NOR) task can be caused by multiple underlying causes, it can indicate changes in the functional connectivity of the hippocampus and the medial prefrontal cortex (mPFC) and requires the animal to distinguish a novel object from a familiar object. Analysis of this preference for novelty in NOR testing showed a significant group effect as indicated by a reduced DI [F (4, 51) = 2.897, *P* = 0.03]. Multiple comparison testing showed that exposure to 30 cGy ^48^Ti significantly reduced recognition memory (*P* = 0.03), while 5 cGy ^48^Ti or ^16^O and 30 cGy ^16^O had no effect on memory retention (Fig. 1a). Following the NOR task, mice were habituated and tested on the object in place (OiP) task which is also dependent on intact hippocampal and prefrontal and perirhinal cortical functions. In this case, functionally intact mice exhibit a preference towards objects that have been moved to a novel location. The results of this test show statistically significant group difference, again indicated by a markedly reduced preference to explore novelty [F (4, 42) = 3.427, *P* = 0.02] (Fig. 1b). Individual analysis showed that ^48^Ti 30 cGy irradiation impaired OiP memory (*P* < 0.004), while 5 cGy ^48^Ti or ^16^O and 30 cGy ^16^O again had no effect on spatial memory function. Lastly, the mice were tested on the temporal order (TO) task where animals were familiarized with two sets of objects, 4 hours apart. Mice with intact hippocampal function show a preference for the first object explored rather than the more recent object. The results of this test demonstrate that cosmic radiation exposure impaired recency memory as reflected by a reduced preference for less recently presented object (Fig. 1c). Analysis of recency discrimination in the TO task revealed an overall group effect [F (4, 43) = 7.753; *P* < 0.0001]. Further multiple comparison analyses showed that all HZE particle exposures impaired recency memory, 5 and 30 cGy ^16^O (*P* < 0.0001 and *P* = 0.01, respectively) and 5 and 30 cGy ^48^Ti (*P* = 0.005 and *P* = 0.05, respectively). Total exploration times of mice for each of these tasks is provided in Supplementary Table 1 (S1A–C). The inability of these irradiated animals to react to novelty after exposure to space relevant doses of cosmic radiation demonstrates the persistence of cognitive decrements in learning and memory. These data extend our past findings obtained at 6 weeks post exposure^16^, and demonstrate that charged particle irradiation results in robust and persistent deficits in recognition and temporal order memory 12 weeks later.

To determine further the extent and nature of cosmic radiation-induced behavioral deficits, a different rodent model (male Wistar rats) was used to assess executive function through their capability to perform attentional set shifting^17^. For these studies, exposure to GCR relevant low doses (5 cGy) of ^48^Ti caused significant decrements (*P* = 0.048, Mann-Whitney) in compound discrimination (CD) when assessed at the same 12 week post-irradiation time point (Fig. 1d). Deficits in CD, indicated by the increased number of attempts required to successfully reach criterion (6 consecutive accurate choices) indicate the relative inability of those animals to identify and focus on task relevant cues. Other tasks involving simple discrimination (SD) and compound discrimination reversal (CDR) were not impaired at this post-irradiation time. These results extend our cognitive findings considerably and now demonstrate that charged particle exposure compromises executive function and temporal order memory that can be linked to impaired perirhinal cortex function over extended post-irradiation times.

**Figure 2:**
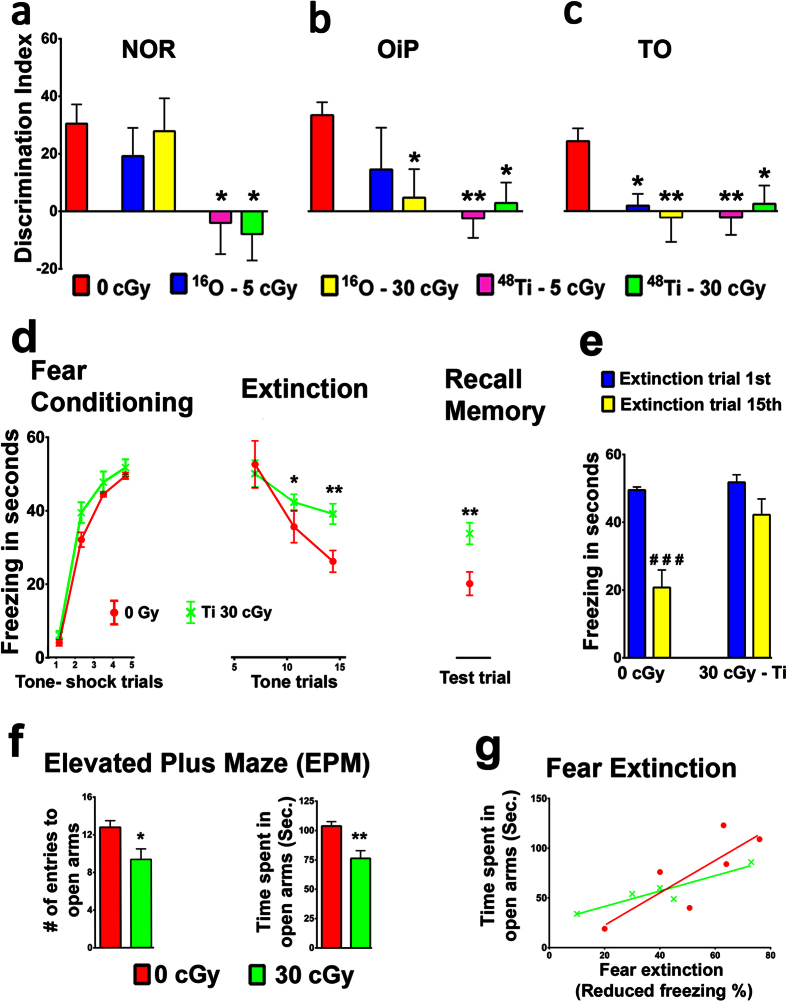
Cognition remains significantly impaired 24 weeks following exposure to cosmic radiation. (**a**) Analysis of the preference for novelty in the **N**ovel **O**bject **R**ecognition (NOR) task demonstrates that charged particle irradiation continues to impair object recognition memory 6 months following exposure to low doses of ^48^Ti particles, while animals exposed to ^16^O remain unaffected. (**b**) Performance on the **O**bject **i**n **P**lace (OiP) task, however, shows significant decrements in spatial memory retention following exposure to 30 cGy ^16^O, and 5 and 30 cGy ^48^Ti particles when compared to controls. (**c**) Analysis of preference for the **T**emporal **O**rder (TO) task shows all ^48^Ti and ^16^O irradiations significantly impaired recency memory as shown by a reduced preference for the less recently explored object. (**d**) Irradiation using 30 cGy of ^48^Ti particles did not impair the acquisition of conditioned fear as demonstrated by similar freezing times observed by tone-shock trial 5 for both control and exposed mice. All mice showed a gradual decrease in freezing behavior over the extinction training on day 3, however the time spent freezing was significantly greater for the irradiated mice as compared to controls. Control mice successful abolish fear memory as demonstrated by reduced freezing behavior in the memory retrieval test when compared to irradiated mice. (**e**) Irradiated mice showed robust freezing between the first and last extinction training session as compared to controls, demonstrating that irradiated mice have a compromised ability to relearn. (**f**) **E**levated **P**lus **M**aze (EPM) testing reveals that charged particle irradiation enhances anxiety-like behavior as demonstrated by reduced numbers of entries and time spent to open arms when compared controls. (**g**) Irradiated mice exhibiting severe extinction impairment also had increased anxiety as compared to control mice that were able extinguished fear memories. *P < 0.05, **P < 0.01, ***P < 0.001, one-way ANOVA followed by Bonferroni’s multiple comparison post hoc analysis; ^###^*P* = 0.001 compared to 1^st^ extinction training, paired t test.

Given the wide-ranging cognitive deficits found 12 weeks after cosmic radiation exposure, we assessed the persistence of these decrements 24 weeks after irradiation (Fig. 2). Analysis at 24 weeks revealed overall significant group differences for the preference of novelty for the NOR task [F (4, 39) = 3.601, *P* = 0.02]. ^48^Ti particle irradiation was again most damaging, significantly reducing the DI of the 5 and 30 cGy irradiated mice, as compared to controls (*P* = 0.03), while neither dose of ^16^O had an effect on recognition memory (Fig. 2a). Performance on the OiP task showed significant decrements in spatial memory retention, again indicated by a markedly reduced preference to explore novelty [F (4, 38) = 5.018, *P* = 0.002]. Multiple comparison analysis showed that the preference for novelty in OiP was significantly reduced following 30 cGy ^16^O (*P* = 0.02) 5 and 30 cGy ^48^Ti (*P* = 0.001 and *P* = 0.02, respectively) irradiation when compared to controls (Fig. 2b). At 24 weeks, HZE particle exposure was again shown to impair TO memory as demonstrated by reduced recency discriminations [F (4, 44) = 5.012, *P* = 0.002] following irradiation. Multiple comparison testing showed that exposure to cosmic radiation significantly reduced preference for the less recently presented, sample phase 1 object, 5 and 30 cGy ^16^O (*P* = 0.04 and *P* = 0.007, respectively) and 5 and 30 cGy ^48^Ti (*P* = 0.003 and *P* = 0.05, respectively; Fig. 2c). Total exploration times of mice for each of these tasks is again provided in Supplementary Table 2 (S2A–C). These data suggest that the effects of irradiation on cognition persist over extended times with no apparent reduction in severity. These longer-term changes in performance were again found to manifest dose independence, suggesting an absence of, or a lower dose threshold (≤5 cGy) for charged particle-induced cognitive impairment. These data demonstrate that space-relevant fluences of charged particles can elicit surprisingly long-term cognitive decrements in learning and memory that persist for at least 6 months.

To ascertain whether cosmic radiation exposure was associated with additional behavioral consequences, a separate cohort of animals was tested for fear extinction and anxiety 24 weeks following exposure to ^48^Ti particles (30 cGy, Fig. 2d,e). Fear extinction refers to an active process that involves the dissociation of a learned response to a prior adverse event. Irradiation had no effect on associative learning as indicated by robust freezing following exposure [Fig. 2d; Two-way RM ANOVA, F (1, 16) = 0.9210, *P* = 0.4]. Unirradiated mice exhibited a gradual decrease in freezing behavior over the extinction training [Fig. 2d, Two-way RM ANOVA, F (1, 18) = 14.61, *P* = 0.001]. Furthermore, time spent in freezing was statistically indistinguishable between control and irradiated groups on day 3 (sessions 11–15) [Multiple t test, DF = 12, *P* = 0.001]. Control mice showed abolished fear memory as demonstrated by reduced freezing behavior in the memory retrieval test when compared to irradiated animals [Fig. 2d, Unpaired t test, DF = 12, *P* = 0.02]. Unirradiated mice exhibited significant extinction or re-learning as evidenced by reduced freezing between first to last (15^th^) extinction training session [Fig. 2e Paired t test, t = 5.035, DF = 6, *P* = 0.002], while irradiated mice continued to show robust freezing [paired t test, DF = 6, *P* = 0.1] during both 1^st^ and 15^th^ trials. Baseline freezing values for this task are provided in Supplementary Table 3 (S3).

The ability to dissociate certain events from adverse outcomes (inhibitory learning) over time helps maintain cognitive health by minimizing stress^18^. When irradiation compromises the process of extinction, increased anxiety may result. To quantify anxiety, animals exposed to ^48^Ti particles (30 cGy) were subjected to an elevated plus maze (EPM) that provides animals the choice of remaining in either “open” or more protected, “closed”, arms of the maze. HZE particle irradiation enhances anxiety-like behavior as evident by reduced numbers of entries into the open arms [Fig. 2f, Unpaired t test, DF = 20, *P* = 0.01] and less time spent in the open arms [Unpaired t test, DF = 16, *P* = 0.001] for the irradiated mice as compared to the control group. Correlations between memory extinction and EPM data show the relationship between heightened anxiety and reduced rates of extinction (Fig. 2g). The ratio of the number of entries and time spent in closed arms versus open arms are provided in Supplementary Table 4 (S4). These data demonstrate that increased anxiety may also contribute to the inability to properly engage “unlearning” processes, representing yet another cognitive risk factor associated with cosmic radiation exposure.

### Cosmic radiation exposure reduces the dendritic complexity of mPFC neurons

**Figure 3:**
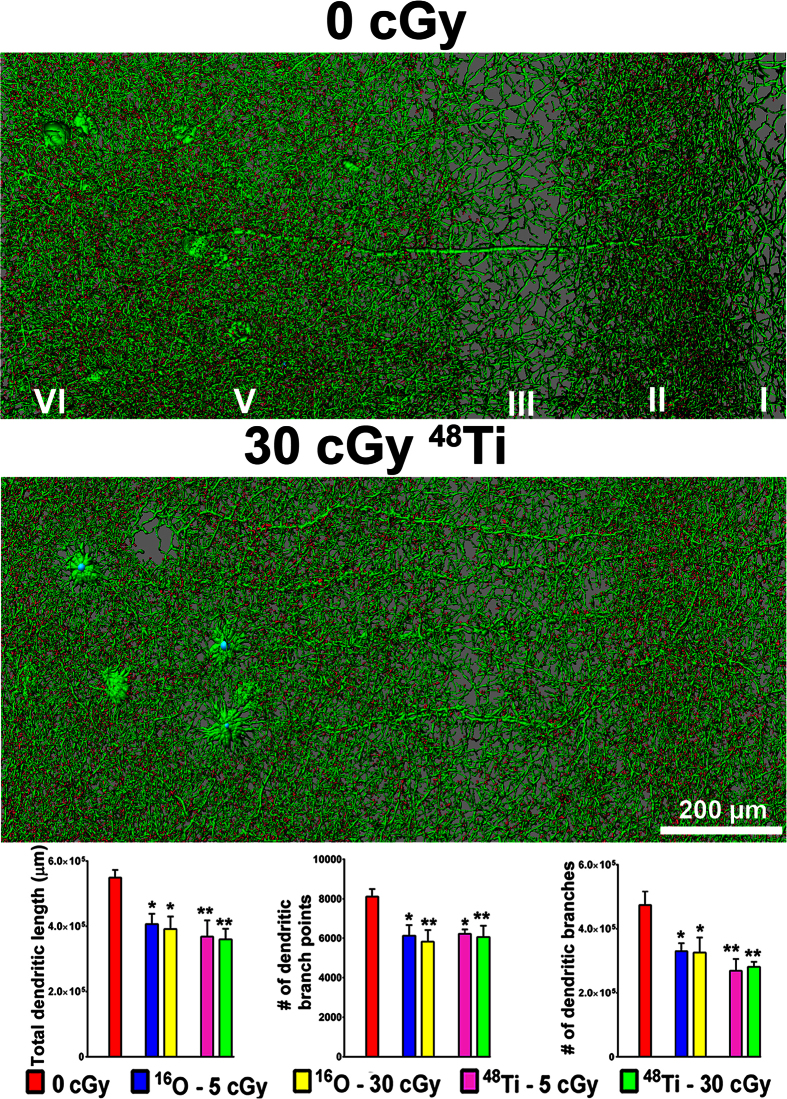
Reduced dendritic complexity of neurons in the prelimbic layer of the mPFC 15 weeks following exposure to cosmic radiation. Digitally reconstructed EGFP-positive neurons from control and irradiated mice showing dendrites (green) and spines (red). Quantification of dendritic parameters (bar charts) shows that dendritic branching and length are significantly reduced 15 weeks after exposure to 5 or 30 cGy of ^16^O or ^48^Ti particles. Data are expressed as mean ± SEM. *P < 0.05, **P < 0.01, ***P < 0.001; one-way ANOVA followed by Bonferroni’s multiple comparison post hoc analysis.

Cognitive changes may be predictive of structural alterations, and based on the behavioral paradigms used we postulated that neurons within the mPFC would exhibit indications of radiation-induced damage. Therefore, following cognitive testing, the morphometric assessment of neurons in the prelimbic layer of the mPFC was conducted. This analysis was facilitated by the enhanced green fluorescent protein (EGFP) expressed in neurons of the Thy1-EGFP transgenic strain of mice, allowing for high resolution imaging of select neurons throughout the brain (14, 15). Digital reconstructions of confocal Z-stacks exhibited marked reduction in the dendritic arborization (Fig. 3, green) of mPFC neurons throughout the prelimbic cortical layers (I–VI) 15 weeks after exposure to charged particles. Quantitative analysis of dendritic branching patterns and length showed significant reductions in the number of dendritic branches, branch points, and overall dendritic length for every dosing paradigm used (Fig. 3). As was found for the cognitive endpoints, none of these long-term structural changes were dose-responsive, suggesting either lower dose thresholds or the absence thereof. Again, these data validate our past findings observed at 6 weeks post exposure^7^, and further demonstrate the marked and persistent deterioration of neuronal structure following cosmic radiation exposure.

### Cosmic radiation exposure reduces spine density along mPFC neurons

**Figure 4:**
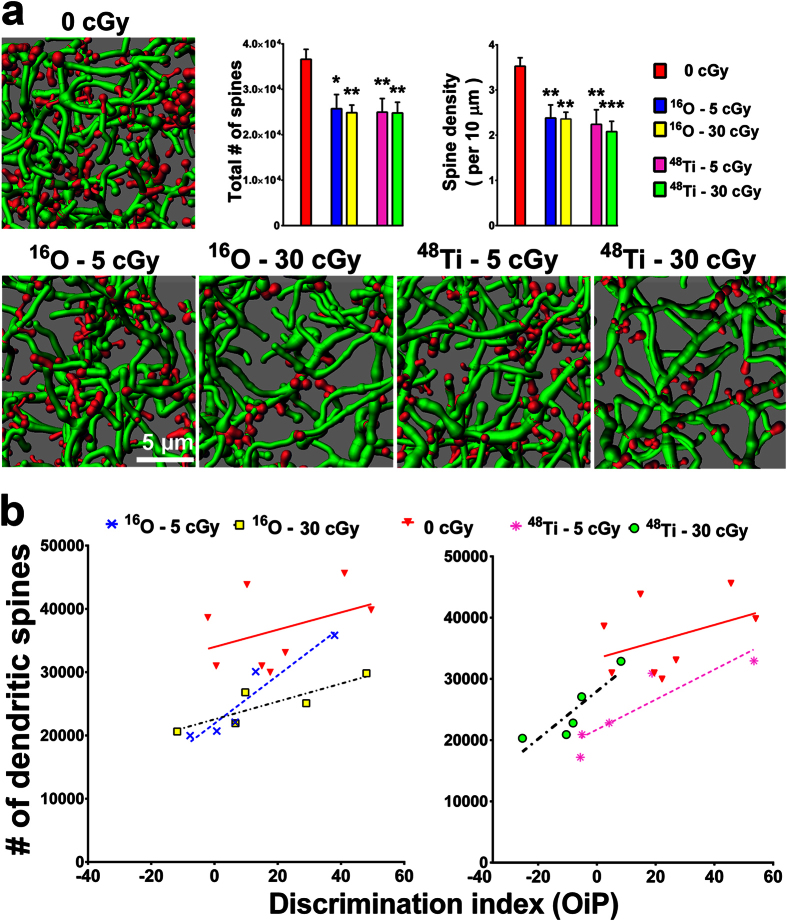
Reduced dendritic spine density in the mPFC 15 weeks following exposure to cosmic radiation. (**a**) Representative digital images of 3D reconstructed dendritic segments (green) containing spines (red) in unirradiated (top left panel) and irradiated (bottom panels) brains. Multiple comparisons show that total spine numbers (left bar chart) and spine density (right bar chart) are significantly reduced after exposure to 5 or 30 cGy of ^16^O or ^48^Ti particles. Data are expressed as mean ± SEM. *P < 0.05, **P < 0.01, ***P < 0.001 versus control; ANOVA. (**b**) Memory deficits correlate with reduced spine density in irradiated mice. Dendritic spine density (per 1.2 mm^2^) is plotted against the corresponding performance of each animal on the OiP task. Radiation-induced reductions in spine number correlate with reduced DI for novelty after exposure to ^16^O (5 cGy, *P* = 0.01; left panel) and ^48^Ti (5 and 30 cGy, *P* = 0.01; right panel).

To determine the effects of cosmic radiation exposure on dendritic spines, higher-resolution analysis of reconstructed dendritic segments was performed. Comparison of control animals to animals exposed to charged particle radiation showed a marked reduction in dendritic spines 15 weeks later (Fig. 4a, red). When normalized to dendritic length, (*i.e.* 10 μm), each charged particle type and dose was found to elicit reduced yields of total dendritic spines and spine density that were dose-independent (Fig. 4a). Consistent with our past results^7^, our present findings highlight yet another structural parameter of neurons that remain compromised at protracted post irradiation times.

### Functional and structural correlations after cosmic radiation exposure

To validate the functional relevance of morphometric analyses, the individual behavioral performance of each mouse (*i.e.* DI value) was plotted against its respective spine density (1.2 mm^2^) for all irradiation paradigms. Correlating dendritic spine density against the corresponding performance of each animal subjected to the OiP task revealed consistent and significant trends (Fig. 4b). For ^16^O exposures, Spearman correlations are as follows: r = 0.29 for 0 Gy controls (*P* = 0.48), r = 1.0 for 5 cGy (*P = 0.01) and r = 0.90 for 30 cGy (*P* = 0.08). For ^48^Ti exposures, Spearman correlations are as follows: r = 0.29 for 0 Gy controls (*P* = 0.48), r = 1.0 for 5 cGy (**P = 0.01) and r = 1.0 for 30 cGy (**P = 0.01). With the exception of animals subjected to 30 cGy ^16^O particles, reduced spine density was correlated significantly with lower DI values for all irradiated cohorts when compared to controls. These data clearly demonstrate the importance of correlating persistent radiation-induced changes in neuronal morphometry to behavioral performance, where certain structural changes in neurons correspond to select deficits in cognition.

### Cosmic radiation exposure reduces specific spine types

**Figure 5:**
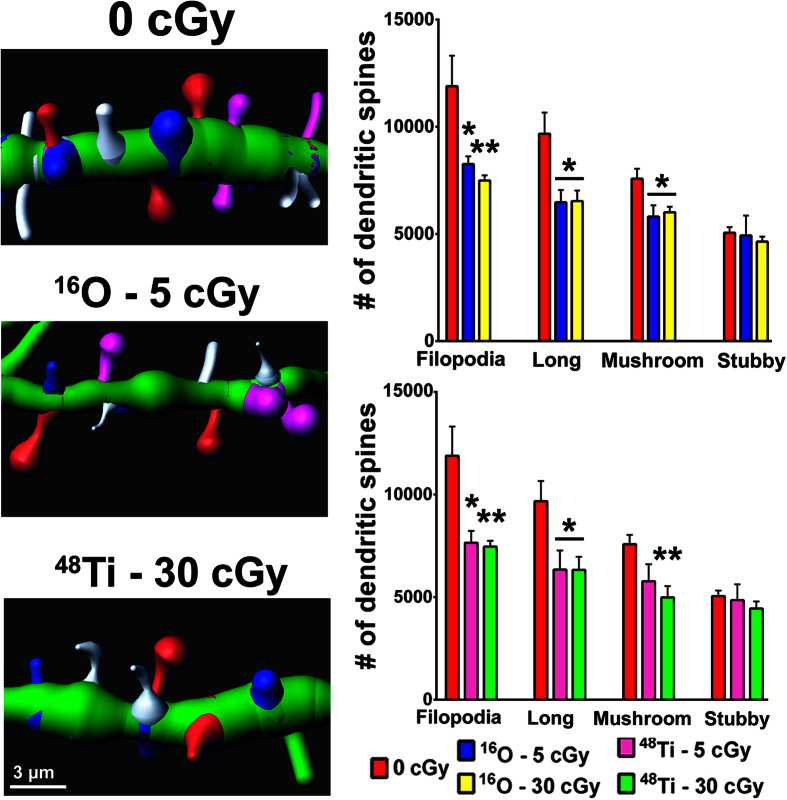
Differential radiosensitivity of dendritic spines. Representative dendritic segments (green) showing immature filopodia (white), long (pink) and mushroon (red) spine types along with more mature stubby (blue) spines. Exposure to ^16^O (upper panel) or ^48^Ti (lower panel) particles leads to significant reductions in the number of immature spines with no effect on mature spines. Quantification of each morphological type of dendritic spine are expressed as the total number of spines for each class. Data are expressed as mean ± SEM. *P < 0.05, **P < 0.01 versus control; ANOVA.

To analyze further potential differences in the susceptibility of morphologically distinct spines to cosmic radiation exposure, distinct subclasses of spines were categorized and quantified 15 weeks following irradiation (Fig. 5). Reconstructed dendritic segments were scrutinized for changes in specific subclasses of spines after irradiation, as shown in representative images. Dendritic spines were classified as filopodia, long, mushroom, or stubby based on rigorous morphometric criteria as described previously^19^. The data clearly illustrate that cosmic radiation exposure caused dose-independent reductions in multiple immature spine types along mPFC neurons. Significantly lower numbers of filopodia (30–37%), thin (32–35%) and mushroom (21–34%) spines types were found after ^16^O or ^48^Ti particle exposures, while mature stubby spines were more radioresistant. These data clearly show that spines of defined morphology exhibit differential susceptibility to cosmic irradiation.

### Increased PSD-95 synaptic puncta after irradiation of mPFC neurons

**Figure 6:**
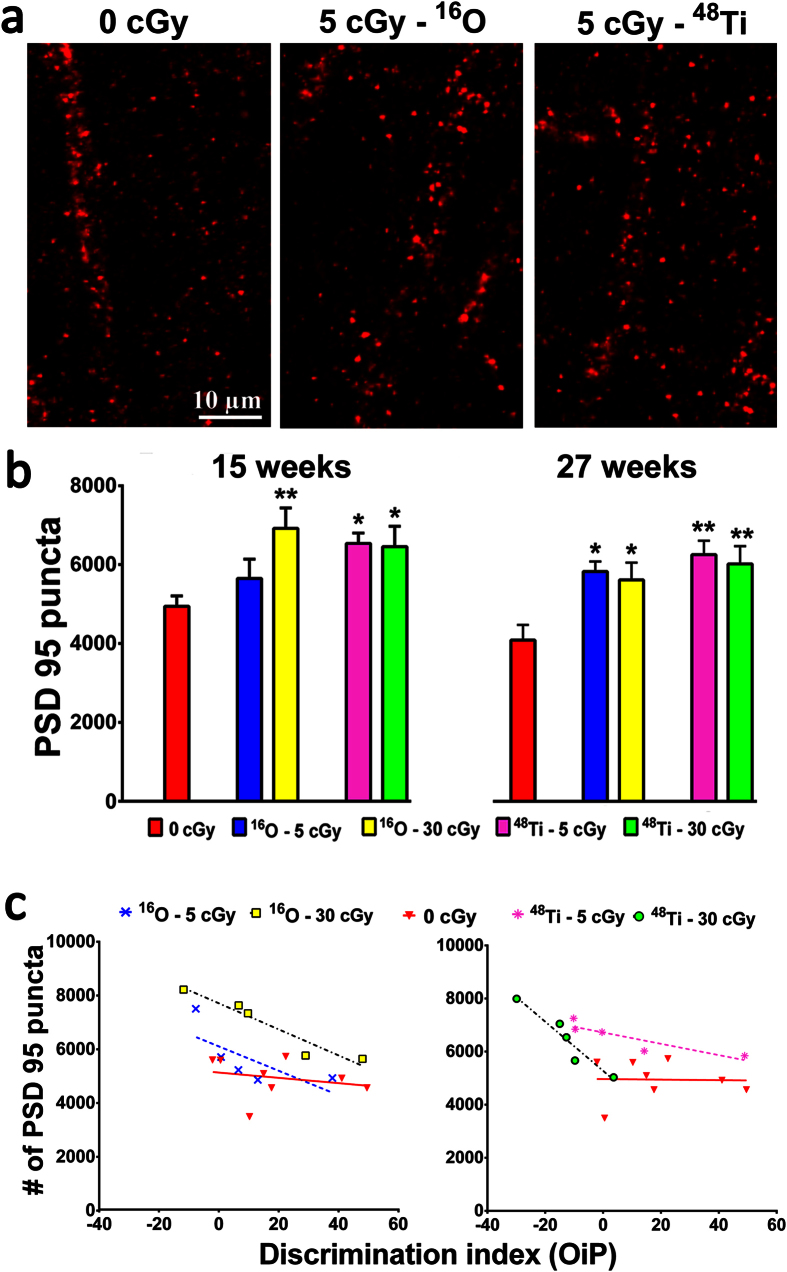
Cosmic radiation exposure induces persistent increases in PSD-95 puncta in the mPFC. (**a**) Representative fluorescence micrographs showing PSD-95 puncta (red). (**b**) Quantitative analyses show that exposure to 5 or 30 cGy of ^16^O or ^48^Ti particles leads to increased numbers of PSD-95 puncta in mPFC neurons as compared to control (15 weeks, left panel; 27 weeks, right panel). Data are expressed as mean ± SEM. *P < 0.05, **P < 0.01, ***P < 0.001 versus control; ANOVA. (**c**) Overexpression of PSD-95 correlates with cognitive decrements in irradiated mice. PSD-95 puncta (per 400 μm^2^) are plotted against the corresponding performance of each animal on the OiP task. Increased levels of PSD-95 puncta are associated with decreased behavioral performance following exposure to ^16^O (30 cGy, *P* = 0.01) and ^48^Ti (5 and 30 cGy, *P* = 0.01).

To complement structural analyses, the levels of postsynaptic density protein 95 (PSD-95) puncta were quantified from deconvoluted confocal images of immunohistochemically stained tissue sections (Fig. 6). High-resolution imaging of brain tissue (layer II of the mPFC) revealed consistent and significant increases in the yield of PSD-95 puncta after all charged particle irradiations (Fig. 6a). Exposure to either dose of ^16^O or ^48^Ti particles increased PSD-95 levels by ~1.2–1.4 fold along neurons in the mPFC in a dose-independent manner (Fig. 6b). These data indicate that, in addition to structural changes, charged particle exposure elicits persistent and significant alterations in the amount and distribution of specific synaptic proteins that remain 15 weeks following acute exposures. Data are also consistent with past data sets obtained at 6 weeks^7^, and indicate that similar irradiation paradigms elicit long lasting changes in critical synaptic proteins.

### Correlating synaptic and functional changes after cosmic radiation exposure

To determine how the observed changes in synaptic PSD-95 puncta corresponded with changes in cognition, similar correlation analyses were performed as those described for spine density (Fig. 6c). For these comparisons, elevated PSD-95 levels significantly correlated with reduced DI for all conditions except those for the animals exposed to 5 cGy ^16^O particles. For ^16^O exposures, Spearman correlations are as follows: r = −0.27 for 0 Gy controls (*P* = 0.48), r = −0.90 for 5 cGy (*P* = 0.08) and r = −1.0 for 30 cGy (**P = 0.01). For ^48^Ti exposures, Spearman correlations are as follows: r = −0.15 for 0 Gy controls (*P* = 0.69), r = −1.0 for 5 cGy (**P = 0.01) and r = −1.0 for 30 cGy (**P = 0.01). Thus, with the exception of animals subjected to 5 cGy ^16^O particles, individual performance on the OiP task was significantly lower and inversely correlated with elevated PSD-95 puncta, an effect that was most pronounced for animals irradiated with 30 cGy of ^48^Ti particles. These data again indicate additional consistent and persistent charged particle-induced effects in the brain. While the functional significance of elevated PSD-95 puncta is less certain, changes in synaptic protein levels have proven to be reliable markers of cosmic and terrestrial radiation exposure of the brain.

### Cosmic radiation-induced neuroinflammation

**Figure 7:**
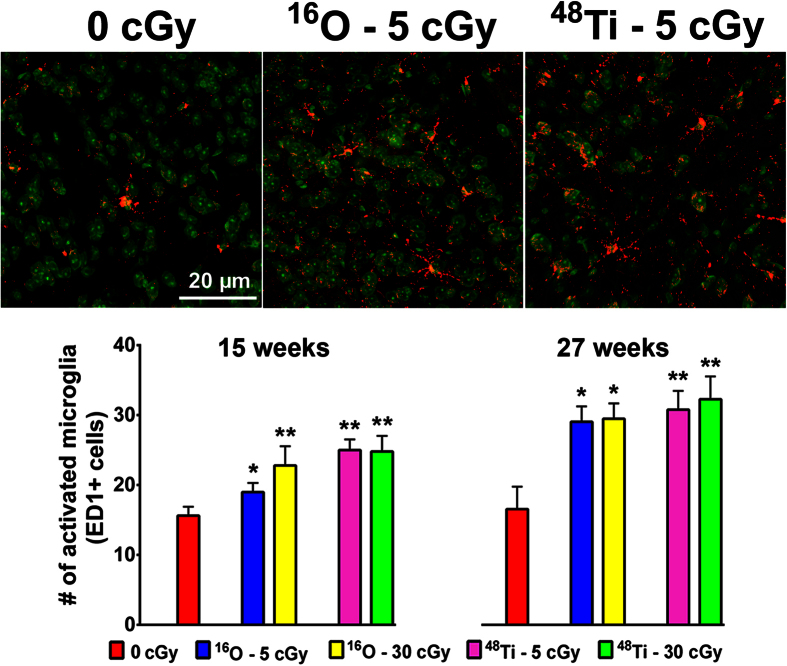
Exposure to cosmic radiation leads to increased neuroinflammation in the prelimbic layer of the mPFC. Representative images from left to right illustrate immunohistochemical visualization of ED-1+ cells in control, 5 cGy ^16^O or 5 cGy ^48^Ti in the PL subfield 15 weeks post-exposure (ED1+ red and toto-3 counterstain; top). Quantitative analysis demonstrates that, compared to respective controls, irradiation leads to increased numbers of activated microglia 15 and 27 weeks later (left and right lower panels, respectively). Data are expressed as mean ± SEM. *P < 0.05, **P < 0.01, ***P < 0.001 versus control; ANOVA.

While morphometric alterations to neurons are likely to play a pivotal role in neurocognitive outcomes, other factors may directly or indirectly impair neurotransmission following cosmic radiation exposure. To ascertain whether charged particle irradiation caused persistent alterations in the levels of activated microglia, ED1 immunopositive cells were quantified 15 and 27 weeks later. For all irradiation conditions and time points, the number of ED1 positive cells increased significantly (Fig. 7). At 15 weeks post-irradiation ED1 levels were increased by ~1.2–1.6 fold and further elevated to ~2-fold at 27 weeks. The persistence of microglial activation is noteworthy and suggests that low dose charged particle exposure elicits an increase in the number of inflammatory cells that prune neuronal processes, thereby disrupting neurotransmission and cognition.

## Discussion

Recent studies from our laboratory have confirmed the adverse effects of cosmic radiation exposure on CNS function^7^,^15^,^16^. Cognitive tasks used in the present study corroborate past findings and identify significant longer-term deficits in episodic, spatial, recognition memory and compound discrimination mapping to regionally defined and more global regions that include the frontal and temporal lobes containing the hippocampus, mPFC and perirhinal cortex^7^,^13^,^14^,^15^,^17^,^20^. Persistent behavioral decrements on the NOR, OiP, TO and attentional set shift testing paradigms lasting 4–6 months following low dose exposure to charged particles, considerably extends our past work^7^ and highlights the exquisite sensitivity of the brain to biophysical interactions with cosmic rays. These new data reveal additional detrimental effects related to fear extinction and anxiety, where charged particle irradiation compromises the ability to dissociate adverse events and outcomes. The inability to moderate reactions to certain unpleasant stimuli could elicit elevated stress, anxiety and otherwise disadvantageous responses in unexpected or emergency situations, responses that have been linked to changes in the mPFC^21^. Such conditions could clearly be problematic for astronauts and their capability to efficiently operate over the course of a deep space mission, and impairments in executive function point to further potential complications in conducting complicated multifaceted tasks or in decision-making under stressful situations. Our new data derived from each of these separate cognitive tests provide convincing evidence of disrupted neurotransmission between cortical and hippocampal circuits in the brain and also suggest that the neurocognitive risks resulting from exposure to cosmic radiation are likely to persist indefinitely.

Understanding precisely how GCR particles damage the brain to elicit such long-term behavioral decrements necessitates knowledge of the frequency and nature of the interaction of these destructive particles with critical brain structures. Highly energetic particles are deeply penetrating, and will produce densely ionizing tracks along particle trajectories as they traverse all cell types in the brain. Delta rays emanating from these primary tracks can elicit secondary sparsely ionizing events as far as 1 cm away, considerably extending the reach of a single particle’s interactions among cells. Taking into account particle dose, linear energy transfer (LET) and fluence/μm^2^ in relation to the geometry of critical neuronal structures, we estimate that the mean number of particle traversals in our study is between 0.25–11.3 for the soma, 2.5–114 for the dendritic tree and 0.013–0.57 for a particular dendritic spine type^22^. These approximations clearly demonstrate that, while direct hits to the soma and dendritic tree are relatively frequent events, those to the spines are not. This also suggests that, if a given particle can ionize targets within a 1-cm cylindrical volume as it traverses the brain, then the probability of any neuronal structure incurring multiple ionizations during a long-term mission to Mars is likely to exceed unity^23^. This estimate may also provide an explanation for the many dose-independent changes observed (*i.e.* cognition, neuronal structure, etc.). If even very low fluences of cosmic particles are predicted to interact with multiple neural cell types and circuits that exhibit unusually high sensitivities, then the overall effect could manifest as an all or nothing response.

Since behavioral decrements can be predictive of structural change, we sought to establish a more conclusive cause and effect by interrogating neurons in those regions of the brain interrogated specifically by our battery of distinct cognitive tasks. Because the mPFC has been implicated as key to complex cognitive behavior including decision-making and executive function and is linked to each of our behavioral tasks^24^, neurons within this region were subject to rigorous structural analyses. In the present study, reductions in dendritic complexity found at 15 weeks after exposure corroborate past findings under similar irradiation conditions conducted at earlier times^7^, and agree with past results obtained in various hippocampal neurons after exposure to different radiation types^16^,^19^,^25^. Importantly, these new data provide additional evidence defining a marked and unexpected persistence of cosmic radiation-induced changes in mPFC neuronal structure. Structural changes were also temporally coincident with increased PSD-95 puncta and activated microglia. While the functional consequences of irradiation on PSD-95 remain uncertain, past work has shown that in immature neurons, overexpression of PSD-95 decreases branching of primary dendrites leading to marked reductions of secondary dendrites^26^. These findings are consistent with present observations (Fig. 5), and suggest that radiation-induced overexpression of PSD-95 inhibits dendritogenesis, with concomitant reductions in the number and density of dendritic spines. Changes in PSD-95 could also disrupt synaptic integrity by altering the composition and distribution of proteins and receptors residing at the synaptic cleft^27^,^28^, and changes in neuroinflammation could actively regulate structural plasticity by pruning dendritic arbors and spines^29^. Thus, given the long-lasting impact of cosmic radiation on neuronal structure, inflammation and synaptic protein levels, it is difficult to envision how these collective processes would not have an adverse effect on neurotransmission and the cognitive well being of those engaged in deep space travel.

To strengthen the causal links between structural and synaptic measurements and cognition, radiation-induced changes in dendritic spines and synaptic puncta were plotted against individual behavior performance on the OiP task. The majority of the data showed significant correlations between either reduced dendritic spine density or increased PSD-95 puncta and lower DI values (Figs 4 and 6). Animal having spine densities below 30,000/0.026 μm^3^ or puncta levels above 6000/0.0018 μm^3^ in the mPFC were likely to exhibit more pronounced behavioral decrements on the OiP task (DI ≤ 10). Collectively this data indicates the importance of preserving the critical structural and synaptic components needed to maintain circuit integrity. Dendritic spines represent the structural correlates of learning and memory and are dependent on the correct stoichiometric balance and organization of critical synaptic proteins^30^,^31^. Therefore, it is not surprising that once spine numbers or synaptic protein levels deviate from their optimal levels, neurotransmission and cognition would be compromised. In a prior report, behavioral testing was found to increase hippocampal spine density, an effect not observed in animals exposed to ^56^Fe particles and subjected to different behavioral paradigms^32^. Thus, while it is possible that behavioral testing can impact spine density and/or synaptic protein levels, in this study, non-irradiated animals exhibited much less variability than found in the irradiated cohorts. Thus, these correlations provide compelling evidence that low dose cosmic radiation exposure elicits defined structural and molecular changes in neurons that are certain to underlie a majority of the marked and persistent neurocognitive side effects observed.

So what does this all mean for a mission to Mars and for NASA’s future plans for deep space exploration? Our recent and current data, along with data from other laboratories, have now clearly identified an unexpected sensitivity of the CNS to cosmic radiation exposure^23^. With this realization comes a concomitant understanding of the risk for developing cognitive deficits that may predispose astronauts to performance decrements, faulty decision-making and longer-term neurodegenerative effects. Given the increased demands, uncertainties and variety of stressors inherent in deep space travel, defining acceptable risk specific to radiation exposure remains a challenge. While carefully controlled terrestrial based experimentation has elucidated a number of potential mechanisms responsible for chronic CNS effects, realistic limitations related to cosmic radiation simulations on Earth and the extrapolation of rodent based behavioral studies to the neurocognitive functionality of astronauts is not without caveats. Furthermore, outbred rodent strains exhibit marked inter-individual variation in the susceptibility to develop HZE-induced cognitive impairment, and the results presented here may not extend to other rodent strains^13^,^14^,^17^,^20^, a situation not uncommon to human subjects. Nonetheless, ground based rodent models are useful for validating and refining assumptions used in various risk assessment models. Rodents have been found to exhibit behavioral parallels with humans on a sequence model of episodic memory^33^ and to possess higher order neuronal structure and sophisticated metacognitive ability thought to be restricted to primates^34^. There are, however, no data that suggest the response of rodent neurons to cosmic radiation would differ in a fundamental way from those of humans, and data derived from rodent-based studies can help identify the types of potential problems that need to be incorporated in any risk management activity related to human error. Thus, the most logical conclusion to draw from these studies is that cosmic radiation exposure poses a real and potentially detrimental neurocognitive risk for prolonged deep space travel. With the growing realization that space is a radioactive environment comes the need to more completely define these risks with more certainty through continued research. Development of more advanced engineering and biologic countermeasures designed to the protect host neuronal circuitry from cosmic radiation exposure are underway, and will certainly be incorporated into deep space mission planning. Unlike humankinds’ other great adventures, space is truly the final frontier. Our exploration of strange new worlds should not be hampered by the fear of cosmic radiation exposure, but rather, inspire robust efforts to advance our understanding of a previously unrecognized problem.

## Materials and Methods

Additional experimental procedures can be found in the Supplementary Materials.

### Animals, heavy ion irradiation, and tissue harvesting

All animal procedures were carried out in accordance with National Institutes of Health and Institutional Animal Care guidelines and were approved by the Institutional Animal Care and Use Committee at the University of California, Irvine and at the Eastern Virginia Medical School. Six-month-old male transgenic mice [strain Tg(Thy1-EGFP) MJrsJ, stock no. 007788, The Jackson Laboratory, Sacramento, CA] harboring the Thy1- EGFP transgene and 7–9 month old male retired breeder (socially mature) Wistar rats (HSD:WI; Harlan Sprague Dawley Inc. Indianapolis, IN) were used in this study. Mice were bred and genotyped to confirm the presence of Thy1-EGFP transgene. Charged particles (^16^O and ^48^Ti) at 600 MeV/n were generated and delivered at the NASA Space Radiation Laboratory (NSRL) at Brookhaven National Laboratory at dose rates between 0.05 and 0.25 Gy/min. Dosimetry was performed, and spatial beam uniformity was confirmed by the NSRL physics staff.

### Behavioral testing

Separate cohorts of mice and rats exposed to cosmic radiation were subjected to 6 distinct behavioral paradigms 12 or 24 weeks after irradiation. Separate cohorts of mice were interrogated using the NOR, OiP and TO tasks at 12 and 24 weeks, while an additional cohort was used for a fear extinction paradigm and EPM task 24 weeks following exposure to quantify additional behavioral performance. Rats were subjected to an attentional set shifting paradigm (12 weeks) designed to interrogate functionality in select cortical regions of the brain including the mPFC, perirhinal cortex and basal forebrain^17^. The NOR, OiP and TO tasks rely on intact hippocampal, mPFC and perirhinal cortex function^35^,^36^. While the NOR task measures the preference for novelty, the OiP task is a test of associative recognition memory and the TO task provides a measure of temporal order memory, that depend on interactions between the hippocampus, mPFC and perirhinal cortices. Spontaneous exploration tasks (NOR, OiP, TO) were conducted as described previously^7^, using the following expression to calculate the DI:


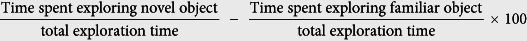


Fear extinction follows a modified fear conditioning protocol^37^ in which repeated trials dissociating the tone-shock pairing can be used to measure the rate of reduced freezing or fear extinction. Deficits in this behavior have been linked to the infralimbic region of the mPFC and require active learning, thereby provide a measure of cognitive flexibility. The elevated plus maze provides a measure of anxiety that can be linked to the amygdala (among other regions), by quantifying the time spent and number of entries into an open versus closed arm of the maze.

Additional details regarding attentional set shifting, fear extinction and EPM testing can be found in the Supplementary Materials.

### Confocal imaging, and neuronal morphometry and spine parameters

The expression of EGFP in specific subsets of neurons provides for the high-resolution imaging and quantification of neuronal structure. In previous studies, we demonstrated that the cosmic radiation types reduced dendritic complexity of hippocampal granule cell and mPFC neurons. Here, we have extended our morphometric analyses of neurons in the prelimbic layer of the mPFC to extended post-irradiation times (15 weeks), using the same rigorously defined morphometric and experimental criteria^19^. Briefly, all morphometric parameters and spine density were quantified from reconstructed neurons in a region of interest (1.2 × 1.2 mm^2^) of the prelimbic cortex (bregma 2.80 mm to 1.50 mm). For dendritic analyses, 100 μm thick sections from the prelimbic cortex were prepared for confocal imaging. Three sections per animal were used to generate Z-stacks from five animals using a Nikon Eclipse TE 2000-U microscope (Nikon, Japan). Quantification included the dendritic structure of both apical and basal dendrites of L1 to L6 neurons. An algorithm for tracing dendritic filaments was used to reconstruct the entire dendritic tree, where tracing originates from the soma and terminates near terminal dendritic diameter thresholds. Reconstructed dendritic segments can be analyzed under higher magnification for dendritic spines that can be labeled, manually verified, morphologically categorized, and quantified. All morphometric parameters were validated from an independent series of pilot reconstructions in both manual and semiautomatic modes. Images were then compared for accuracy and consistency to ensure that selected parameters represented actual variations in dendritic structure. Parameters of neuronal structure that were identified and quantified through image reconstruction and deconvolution using the IMARIS software suite (Bitplane Inc.) included the cell body, dendritic and axonal length, branching and branch points, dendritic complexity, spines, and boutons.

The number of dendritic spines was determined by summing total number of spines in the same region of interest, where spine density was calculated by dividing total dendritic length by the total number of spines. Spines were classified based on the following morphological parameters: (i) Stubby spine: Stubby spines are devoid of a neck, diameter of the head is almost equal to the total length of the spine. (ii) Long spine: Length of neck is greater than its diameter and the head is clearly distinguishable but has a diameter less than the length of the neck. (iii) Mushroom spine: Mushroom spines have a large head and a narrow neck, diameter of the head is greater than the width of the neck. (iv) Filopodia spine: Total spine length is greater than 1 μm with the complete absence of a head.

### Immunohistochemistry of synaptic proteins

Coronal sections of the brain were immunostained for the quantification of PSD-95 as described previously^19^.

### Statistical analysis

Data are expressed as mean ± SEM of 4 to 10 independent measurements. The level of significance was assessed by one-way ANOVA along with Bonferroni’s multiple comparison using Prism data analysis software (v6.0). Correlation of spine density or PSD-95 puncta to individual DIs was performed using the Spearman rank test. Statistical significance was assigned at P < 0.05.

## Additional Information

**How to cite this article**: Parihar, V. K. *et al.* Cosmic radiation exposure and persistent cognitive dysfunction. *Sci. Rep.* **6**, 34774; doi: 10.1038/srep34774 (2016).

## Supplementary Material

Supplementary Information
