# Supplementary material for: Cosmic radiation exposure and persistent cognitive dysfunction
Source: Sci Rep. 2016 Oct 10;6:34774. doi: 10.1038/srep34774 (PMC5056393; doi:10.1038/srep34774)
Supplement: Supplementary Information [file srep34774-s1.pdf]

Supplementary Information

Cosmic radiation exposure and persistent cognitive dysfunction

Vipan K. Parihar[1], Barrett D. Allen[1], Chongshan Caressi[1], Stephanie Kwok[1], Esther Chu[1], Katherine K. Tran[1], Nicole N. Chmielewski[1], Erich Giedzinski[1], Munjal M. Acharya[1], Richard A. Britten[2], Janet E. Baulch[1] and Charles L. Limoli[1,*]

[1]Department of Radiation Oncology, University of California, Irvine, CA 92697-2695, USA

[2]Department of Radiation Oncology, Eastern Virginia Medical School, Norfolk, VA 23507

Table S1A: In the **N**ovel **O**bject **R**ecognition (NOR) task, control mice displayed a clear preference for exploring the novel object over the familiar object, an effect not found with the irradiated cohorts. During the test phase, mice spent 68% of their time exploring the novel object.

Treatment	Time Spent Exploring Novel Object (Sec.)	Time Spent Exploring Familiar Object (Sec.)	Total Exploration Time (Sec.)
0 Gy	25.90± 2.6	16.60±2.1	42.50±6.7
^{16}O 5 cGy	19.00±4.3	15.20±2.3	34.20± 5.8
^{16}O 30 cGy	18.00±4.5	12.00±2.1	30.00±5.3
^{48}Ti 5 cGy	14.00± 3.4	13.00±5.5	27.00± 3.1
^{48}Ti 30 cGy	11.30±2.8	12.00±3.5	23.30± 4.4

Table S1B: In the **O**bject **i**n **P**lace (OiP) task, control mice exhibited a strong preference for the objects placed at novel locations. Irradiated groups showed almost no preference for any of the objects and exhibited significant decrements compared with sham-irradiated controls.

Treatment	Time Spent Exploring Objects at Novel location (Sec.)	Time Spent Exploring Objects at Familiar location (Sec.)	Total Exploration Time (Sec.)
0 Gy	34.00± 4.5	24.00±3.6	58.00±4.9
^{16}O 5 cGy	18.00±4.3	16.00±3.9	34.00±4.1
^{16}O 30 cGy	25.50±2.7	23.00±5.6	48.50±4.7
^{48}Ti 5 cGy	16.00±3.3	15.00± 4.5	31.00±7.7
^{48}Ti 30 cGy	20.00±3.9	23.00± 4.7	43.00±6.7

Table S1C: In the **T**emporal **O**rder (TO) task, cosmic radiation exposure impaired recency memory that was evident by the reduced preference for the object presented prior to the last (i.e., sample object 1).

Treatment	Time Spent Exploring Object from sample 1 (less recent) (Sec.)	Time Spent Exploring Object from sample 1 (Sec.)	Total Exploration Time (Sec.)
0 Gy	23.00±2.1	10.50±2.1	33.50±4.3
^{16}O 5 cGy	10.00±3.3	15.00±5.2	25.00±5.4
^{16}O 30 cGy	14.30±3.0	13.80±3.8	28.10±6.3
^{48}Ti 5 cGy	12.50±4.9	13.00±3.9	25.50±4.3
^{48}Ti 30 cGy	14.80±3.9	12.70±3.2	27.50±5.3

Table S2A: Cosmic radiation significantly impairs novel object recognition in mice 24 weeks following irradiation. Irradiated mice showed significantly reduced preference for the novel object as compared to non-irradiated controls.

Treatment	Time Spent Exploring Novel Object (Sec.)	Time Spent Exploring Familiar Object (Sec.)	Total Exploration Time (Sec.)
0 Gy	28.00±3.2	15.00±2.1	43.00±4.1
^{16}O 5 cGy	17.90±3.2	12.00±3.2	29.90±5.3
^{16}O 30 cGy	18.00±4.2	10.20±5.4	28.20±3.0
^{48}Ti 5 cGy	15.00±5.4	16.00±3.3	31.00±3.9
^{48}Ti 30 cGy	15.00±2.1	17.60±3.4	32.60±5.1

Table S2B: Cosmic radiation impaired acquisition and retrieval of object-in-place performance that was evident by a reduced preference for objects at novel locations.

Treatment	Time Spent Exploring Objects at Novel location (Sec.)	Time Spent Exploring Objects at Familiar location (Sec.)	Total Exploration Time (Sec.)
0 Gy	41.00± 3.2	23.00± 2.8	64.00±4.9
^{16}O 5 cGy	28.20±3.3	22.10±5.1	50.30±6.9
^{16}O 30 cGy	32.60±3.2	30.0±4.3	62.60±8.9
^{48}Ti 5 cGy	31.00±4.1	33.00±3.2	64.00±9.8
^{48}Ti 30 cGy	28.00±5.1	30.00±4.2	58.00±7.1

Table S2C: Cosmic radiation impaired recency memory that was evident by the reduced preference for the object presented prior to the last.

Treatment	Time Spent Exploring Object from sample 1 (less recent) (Sec.)	Time Spent Exploring Object from sample 1 (Sec.)	Total Exploration Time (Sec.)
0 Gy	13.00±2.1	8.00±2.1	21.00±5.1
^{16}O 5 cGy	12.00±3.2	11.60±3.2	23.60±7.9
^{16}O 30 cGy	8.50±2.2	9.00±3.2	17.50±7.1
^{48}Ti 5 cGy	9.00±4.3	9.40±4.3	18.40±6.5
^{48}Ti 30 cGy	7.50±3.2	7.20± 4.3	14.70±5.5

Table S3: Baseline freezing (Sec.) during fear conditioning and extinction training

Treatment	Fear Consolidation	Extinction Trial (0-5)	Extinction Trial (6-10)	Extinction Trial (11-15)
0 Gy	4.05±0.82	4.78 ±0.48	4.23±0.56	3.68±0.67
Ti 30 cGy	6.30± 1.08	6.38±0.58	5.26±0.70	4.54± 0.57

Table S4A: The number of entries and ratio of the number of entries (closed:open arms). The effects of cosmic radiation on anxiety were determined using an **E**levated **P**lus **M**aze (EPM). Irradiation enhances anxiety-like behavior as demonstrated by the reduced numbers of entries into open arms when compared to controls.

Treatment	Number of Entries to Closed Arms	Number of Entries to Open Arms	Ratio Time Spent (Close/Open Arms)
0 Gy	19.35±0.94	12.78±0.71	1.51
^{48}Ti 30 cGy	21.37±2.03	9.37 ± 0.85	2.28

Table S4B: The time spent and ratio of the time spent (closed:open arms). Cosmic radiation exposure reduces the preference for the open-arms, as irradiated mice prefer to remain in the darker, closed arms.

Treatment	Time spent in Closed Arms (Sec.)	Time Spent in Open Arms (Sec.)	Ratio Time Spent (Close/Open Arms)
0 Gy	204.53±11.92	103.82± 3.39	1.97
^{48}Ti 30 cGy	217±5.84	76.00±6.5	2.86
